# Investigation of Spark Plasma Sintering on Microstructure-Properties of Zirconia Reinforced Fluormica Glass for Dental Restorations

**DOI:** 10.3390/ma16186125

**Published:** 2023-09-08

**Authors:** Sivaranjani Gali, Suhasini Gururaja, T. Niranjana Prabhu, Srikari Srinivasan

**Affiliations:** 1 Department of Prosthodontics, Faculty of Dental Sciences, M.S. Ramaiah University of Applied Sciences, Bengaluru 560054, India; 2 Department of Aerospace Engineering, Auburn University, Auburn, AL 36849, USA; 3 Department of Chemistry, Faculty of Mathematical and Physical Sciences, M.S. Ramaiah University of Applied Sciences, Bengaluru 560058, India; 4 Department of Automotive & Aerospace Engineering, M.S. Ramaiah University of Applied Sciences, Bengaluru 560058, India

**Keywords:** spark plasma sintering, zirconia, fluormica glass, dental ceramics, dental restorations

## Abstract

Background: Conventional sintering methods of dental ceramics have limitations of high temperature and slow cooling rates with requirements of additional heat treatment for crystallization. Spark plasma sintering (SPS) is an emerging technique that has the potential to process dental restorations with dense microstructures and tailor-made clinically relevant properties with optimized processing parameters. This study explored the potential of the SPS of zirconia-reinforced fluormica glass (FM) for dental restorative materials. Methods: FM glass frit was obtained through the melt-quench technique (44.5 SiO_2_–16.7 Al_2_O_3_–9.5 K_2_O–14.5 MgO–8.5 B_2_O_3_–6.3 F (wt.%)). The glass frit was ball-milled with 20 wt.% of 3 mol% yttria-stabilized zirconia (FMZ) for enhanced fracture toughness. The mixtures were SPS sintered at a pressure of 50 MPa and a heating rate of 100 °C/min for 5 min with an increase in temperature from 650–750 °C–850 °C–950 °C. Phase analysis was carried out using XRD and microstructural characterization with SEM. Micro-hardness, nano-indentation, porosity, density, indentation fracture toughness, and genotoxicity were assessed. Conclusions: The increase in the SPS temperature of FMZ influenced its microstructure and resulted in reduced porosity, improved density, and optimal mechanical properties with the absence of genotoxicity on human gingival fibroblast cells.

## 1. Introduction

The global market of dental restorations shows an increasing demand for restorative materials, implants, and prosthetics. Therefore, advances and innovations in biomaterials must keep pace with the growing patient demands for dental treatment of missing teeth [1]. In the development cycle of dental restorative materials, it is imperative to achieve clinically appropriate properties of dental materials such as aesthetics, strength, biocompatibility, and chemical durability [2,3].

The success of lithium disilicate-based glass-ceramics and zirconia as indirect dental restorations is due to their excellent mechanical properties, chemical inertness, and aesthetics. However, glass-ceramics have limitations of moderate strength, sub-optimal chemical durability, need for two-stage crystallization, and possible deformation of dental restorations during heat treatment [4,5]. Zirconia exhibits low-temperature degradation, opacity, etching difficulties, requires high-temperature sintering with slow cooling rates, and its associated shrinkage post-machining [6,7,8,9].

Due to these challenges with dental glass-ceramics and zirconia, alternate ceramic configurations such as lithium disilicate with 10 wt.% of zirconia were explored [10]. Fluormica (FM) has been used for dental restorations, such as castable glass-ceramics (DICOR) and machinable glass-ceramics (MACOR). In the 1980s, alkaline-free tetrasilicic mica based on a SiO_2_-MgO-K_2_O-F system for dental restorative crowns was researched [11,12,13,14]. They are known for their machinability, cytocompatibility, and chemical durability. With FM-based glass-ceramic dental restorations requiring complex ceramming procedures with reports of low clinical survival rates, molar fractures, and modest mechanical properties, alkali and alkaline earth ion additions to FM-based glasses were attempted to enhance their mechanical properties [15,16,17]. Zirconia, known for its high fracture toughness, was used to reinforce FM-based glass systems using conventional pressureless sintering techniques [18,19,20,21,22,23,24,25,26].

As conventional sintering techniques of dental zirconia and glass-ceramics have limitations of sintering at high temperatures and are time-consuming with slow heating rates, with the requirement of additional heat treatment procedures, advanced sintering techniques such as spark plasma sintering (SPS) have been explored recently [27]. SPS is an emerging sintering process that employs a low-voltage, direct pulsed current-activated pressure. It is a pressure-assisted sintering method that uses pressure, temperature, and current to densify ceramics. This process has the advantages of low sintering temperature, faster heating rates, and short holding times and offers densification under high pressure [28]. Rapid heating rates of SPS hinder undesirable grain growth and promote reduced grain size, achieving higher densification [27,29,30,31].

The SPS technique has been previously used to produce transparent, high-strength, and non-porous dense ceramics [32,33,34,35,36,37,38,39,40]. Transparent nanocrystalline ceramics with superior flexural strength were acquired with SPS sol–gel-based zirconia silica glass [39]. Dense and fine crystalline microstructures were achieved with increased pressure, temperature, and heating rates of SPS-sintered dental lithium disilicate glass-ceramic powders [41]. High flexural strengths with reduced voids were seen in functionally graded ceramics with varying volume fractions of SPS zirconia/porcelain [42]. Enhanced mechanical properties of flexural strength, fracture toughness, hardness, and density were obtained with SPS 20 wt.% zirconia lithium silicate glass-ceramics [43]. SPS soda-lime glass showed high toughness, bending strength, and Weibull modulus with augmented biocide activity [44,45]. Fine crystalline microstructures of 20 wt.% zirconia-bearing lithium silicate glass were obtained with increased SPS sintering temperatures [46]. Dense and amorphous microstructures with improved bioactivity were observed in SPS Bioglass 45S [47]. SPS of mica-based glass for dental restorations improved its machinability, with an increase in the percentage of the fluorphlogopite phase from 57% to 61% [48]. SPS was investigated for titanium-based dental implants for their bio-corrosion, biocompatibility, and mechanical properties [49,50].

Based on the above literature, most studies have investigated SPS-sintered alkaline and alkaline earth silicate, oxynitride, aluminosilicate, sol–gel-based zirconia–silica, and soda-lime glass systems. Investigation of SPS on FM glass systems with zirconia addition has not been attempted so far. The current work examines the effect of SPS sintering temperatures on microstructure and properties of zirconia-reinforced FM glass (abbreviated as FMZ) to develop dense and tough ceramics for dental restorations.

## 2. Materials and Methods

### 2.1. Glass Preparation

Precursor powders of the FM-based glass composition (44.5 SiO_2_–16.7 Al_2_O_3_–9.5 K_2_O–14.5 MgO–8.5 B_2_O_3_–6.3F (wt.%)) in Table 1 were ball-milled at 300 rpm for 6 h to ensure homogenous mixing. The ball-milled glass powder was melted in a platinum crucible at 1500 °C in an electrical furnace and quenched in cool, deionized water until room temperature to obtain glass frit. Based on the previous studies, 20 wt.% of YSZ was added to enhance the fracture toughness of FM glass [18,19]. The glass frit powder (D_50_~3.08 µm) was further ball-milled with 20 wt.% of 3 mol% yttria-stabilized zirconia (YSZ, Tosoh, Japan) (D_50_~50 nm) for homogeneity. The mixture was SPS in a 20 mm graphite die (SPS-7.40MK-VI, Fuji Electronic Industrial Co., Ltd., Saitama, Japan) under 50 MPa compressive stress with a heating rate of 100 °C/min at sintering temperatures of 650 °C, 750 °C, 850 °C, and 950 °C for 5 min. SPS-sintered FM glass with 20 wt.% YSZ will be abbreviated as SPS-FMZ and SPS-FM glass without 20 wt.% YSZ as SPS-FM throughout the paper.

### 2.2. Sintering Method

The glass transition temperature (T_g_) and crystallization temperature (T_c_) of the ball-milled glass powder were previously determined using a differential scanning calorimeter (DSC) and were found to be 740 °C and 1060 °C, respectively. The temperatures required for SPS are usually about 200–250 °C less than conventional sintering [51]. Further, high temperatures (more than 950 °C) during SPS had the risk of excessive flow and loss of glass [41]. Hence, in the present study, the temperature range of 650–750–850–950 °C was followed for SPS-FMZ. The heating rate of 100 °C/min with a pressure of 50 MPa was based on the previous literature [41]. At a constant pressure of 50 MPa and heating rate of 100 °C/min for 5 min, the influence of an increase in temperature from 650 °C, 750 °C, 850 °C, to 950 °C on phase assemblage, density, porosity, microstructure, and mechanical properties of SPS-FMZ was investigated. The following sections describe the materials used for this study and the methodology of preparation of FMZ glass using spark plasma sintering with characterization methods and genotoxicity tests.

### 2.3. Phase Analysis and Microstructural Characterization

Phase analysis of sintered specimens using X-ray Diffraction (Bruker D8 ADVANCE X-ray Diffractometer, Malvern, UK) 2.2 kW X-ray source of Cu anode operated at 40 kV Voltage and 40 mA current at 1.6 kW power with a step size of 0.026°) was carried out to determine major and minor phases. The specimens were polished with silicon carbide papers (SiC) under water cooling. Polishing was conducted on the top surfaces using 0.5 µm diamond paste and colloidal silica to obtain an acceptable optical surface for viewing under SEM. The sintered SPS-FMZ specimens were etched using 12% hydrofluoric acid for 3 min. Etched specimens were gold-coated using a sputter coater (Quorum Technologies, Laughton, UK) for 30 s with 40 mA. SEM (Schottky Field Emission, Gemini Column, Oberkochen, Germany) with energy dispersive spectroscopy was used for microstructural analysis.

### 2.4. Density and Porosity Characterization

Apparent densities of sintered SPS-FMZ specimens were measured by the Archimedes method based on the relative differences between the weights of the specimens in air and distilled water. Pore size distribution of the sintered specimens was measured by mercury intrusion porosimetry (POREMASTER 60, Quantachrome Instruments, Gurugram, Haryana, India). Percentage porosity was calculated as the ratio of intruded volume and total volume.

### 2.5. Mechanical Characterization

The methodology for SPS-FMZ sintered ceramic’s hardness and indentation fracture toughness was adopted from the previous literature on dental ceramics [18].

#### 2.5.1. Micro-Hardness

The Vickers indentation test was used to evaluate the micro-hardness of each of the sintered SPS-FMZ discs of 20 mm diameter and 2 mm thickness. The methodology followed was based on the previous literature and ASTM E92 [18,19]. The polished surfaces were indented using a micro-hardness tester (n = 5 indentations) (Wilson^®^ VH1102, Buehler, IL, USA) with an indent load of 10 N with a dwell time of 10 s. The indent diagonals were measured using an optical microscope using the following formula (Equation (1)), where P is the indentation load (N) and d is the average diagonal length (mm) [52].

(1) $$H_{v}=\frac{1.85P}{d^{2}}$$

#### 2.5.2. Nano-Indentation

Nano-indentation testing was performed at room temperature in ambient air (23 °C) using a nano-indenter (Agilent, G200 series, Santa Clara, CA, USA) equipped with a Berkovich diamond three-sided pyramid. A loading profile was developed with a peak load of 10 mN, a peak hold time of 10 s with 100% unload, and 15 s of time to load with an allowable drift rate of 0.05 nm/s. During nano-indentation, forces and displacements were recorded to generate load–displacement (P-h) curves. Hardness, H (n = 10 indentations), was determined from the following relation (Equation (2)):

(2) $$H=\frac{P}{A}$$

where P is the maximum applied load and A is the indentation area. During the indentation process, obtained P-h curves were used to determine reduced elastic modulus (E*) based on the Oliver–Pharr method [53].

#### 2.5.3. Indentation Fracture Toughness

The Vickers indenter made indentations at a load of 49 N (n = 5 indentations). Crack lengths were measured using SEM, and the mode I fracture toughness (K_IC_) was calculated using the following formula in Equation (3). Here, P is the indentation load (N), H is hardness (GPa), E is elastic modulus (GPa), and c is the average crack length (µm) [54].

(3) $$K_{IC}=0.016\;\;{\left(\frac{E}{H}\right)}^{1/2}\;\frac{P}{c^{3/2}}$$

### 2.6. Comet Assay for Genotoxicity

SPS-FMZ specimens were sterilized (70% ethanol for 10 min and Under UV for 10 min) and incubated in Dulbecco’s Modified Eagle’s Medium (DMEM)-high glucose medium for 24 h at 37 °C and 5% CO_2_. Human gingival fibroblast cells were cultured in a 6-well plate at a density of 10 × 10^5^ cells/0.2 mL and incubated in a CO_2_ incubator overnight at 37 °C for 12 h. The spent medium was aspirated and washed with 1 mL of 1X Phosphate-Buffered Saline (PBS). Cells were treated with the concentration mentioned above of the sintered SPS-FMZ ceramic specimen (DMEM) with leached components of the test material, and cells were incubated for 24 h. The spent media were removed, and the concentration mentioned above of sintered ceramic specimens in 2 mL of culture medium was added and cells were incubated for 24 h.

Further, the cells were mixed with low melting agarose in a 1:1 dilution, and 85 µL of the concentration of the sintered specimens was placed on the comet assay slides. Cells were lysed with lysis buffer through overnight incubation at 4 °C. Cellular proteins were removed from the cells through lysis. Under alkaline/neutral conditions, DNA was allowed to unwind and undergo electrophoresis, allowing the broken DNA fragments or damaged DNA to migrate away from the nucleus. DNA-specific fluorescent dye such as ethidium bromide was used for staining, and the amount of fluorescence in the head and tail and the tail length were determined using a fluorescent microscope. The extent of DNA liberated from the head of the comet is directly proportional to the amount of DNA damage. The presence of tails, appearing as comets, indicates damaged cells, whereas the absence of tails with intact nuclei indicates undamaged cells.

### 2.7. Statistical Analysis

Statistical Package for Social Sciences (SPSS, Windows Version 22.0 Released 2013. Armonk, NY, USA, IBM Corp.) was used for statistical analysis. Descriptive analysis included expression of hardness and reduced elastic modulus in terms of median and interquartile ranges due to the wide scatter of the respective values. Mean and standard deviation were used for fracture toughness. Inferential statistics included a one-way ANOVA test followed by Tukey’s post hoc test to compare the sintered groups. The level of significance was set at *p* < 0.05.

## 3. Results

**Figure 1:**
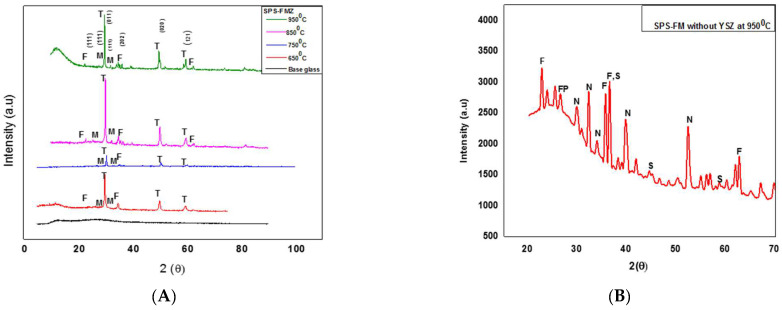
XRD plots. (**A**) SPS-FMZ of base glass with 20 wt.% YSZ at temperatures of 650 °C, 750 °C, 850 °C, and 950 °C revealing tetragonal (T) and monoclinic (M) phases of YSZ with minor peaks of forsterite (F) and (**B**) SPS-FM without YSZ at 950 °C showing minor phases of forsterite (F), fluorophlogopite (FP), norbergite (N), and spinel (S).

The results of the phase assemblage of SPS-FMZ sintered at 650 °C–750 °C–850 °C–950 °C are presented in Figure 1. XRD patterns of FM-based glass reveal a broad and diffuse peak in the 2θ range between 10 and 40° in Figure 1A, confirming the amorphous nature of glass. SPS-FMZ at 650 °C, 750 °C, 850 °C, and 950 °C showed major crystalline peaks of tetragonal and monoclinic phases of YSZ with amorphous glass. The presence of the tetragonal phase of YSZ (T) at 2θ values of 30.2°, 50°, and 60° (011, 020, and 121) and the monoclinic phase of YSZ (M) at 2θ values of 28.2° and 31.5° (111) was confirmed by the sharp peaks of SPS-FMZ. Minor peaks of forsterite (F) (111 and 202) at 2θ values of 35.8°, 22.9°, 36.6°, and 62° were observed. SPS-FM glass without YSZ revealed minor peaks of forsterite (F), fluorophlogopite (FP), norbergite (N), and spinel (S), as seen in Figure 1B.

**Figure 2:**
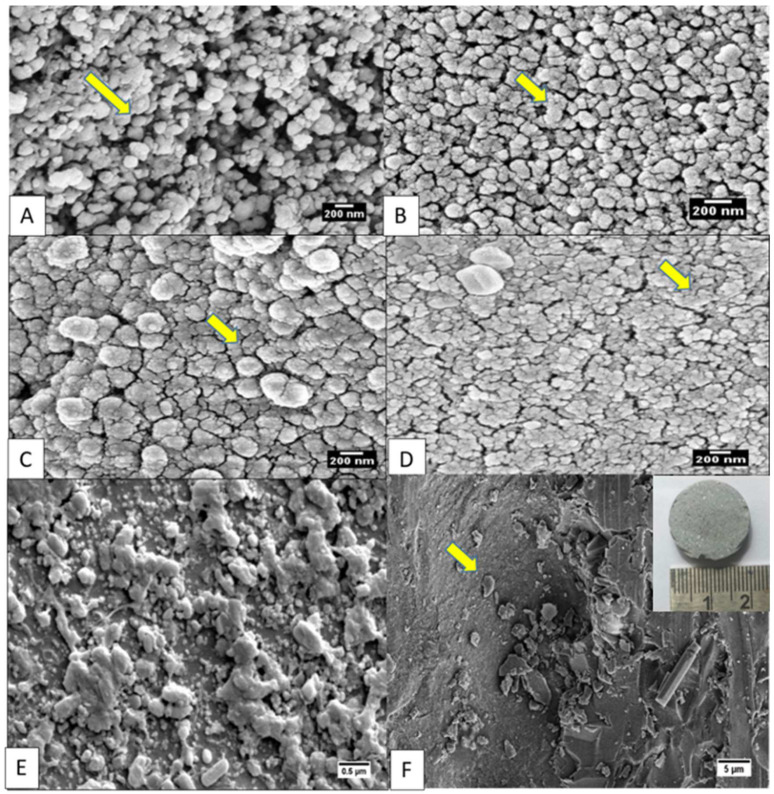
Scanning electron images (SEM). SPS-FMZ of base glass with 20 wt.% YSZ at temperatures of 650 °C (**A**) with non-reactive spherical particles (arrow), at 750 °C (**B**) with necking of particles (arrow), at 850 °C (**C**) with fusion of particles (arrow), and at 950 °C (**D**) with coalescence of particles (arrow), (**E**) SPS-FM without YSZ at 950 °C, and (**F**) fractured surface of SPS-FMZ at 950 °C exposing a porosity on the unetched surface (arrow) and the inset image shows the SPS-FMZ sample.

**Figure 3:**
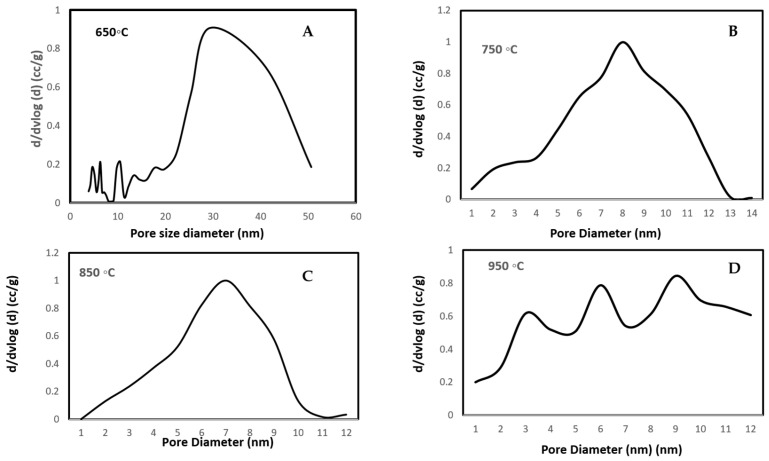
Pore size distribution of SPS-FMZ at temperatures of (**A**) 650 °C, (**B**) 750 °C, (**C**) 850 °C, and (**D**) 950 °C at a constant pressure of 50 MPa.

**Figure 4:**
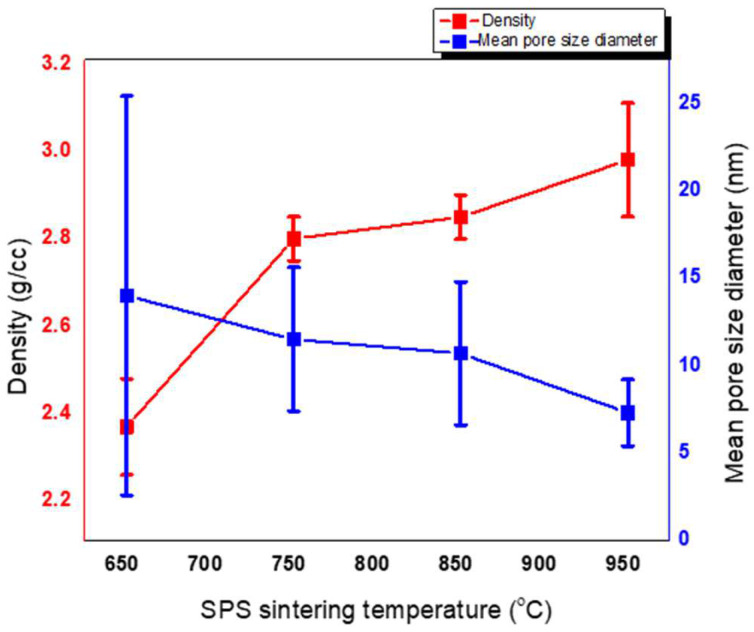
Effect of sintering temperature on density and mean pore size diameter of SPS-FMZ.

Microstructural evolution with sintering temperature is shown in Figure 2A–D. At 650 °C, the microstructure shows spherical particles with visible pores located between the particles. At 750 °C, widespread necking of particles could be seen. With further increases in temperature to 850 °C and 950 °C, the coalescence of particles into spherical agglomerates was observed. The microstructure of SPS-FM without YSZ can be seen in Figure 2E. The fractured surface of SPS-FMZ at 950 °C exposing porosity can be seen in Figure 2F and the insert image in Figure 2F shows the SPS-FMZ sample. Density values with porosity are presented in Table 2 and pore size distributions are in Figure 3A–D. The effects of an increase in SPS sintering temperatures on density and mean pore size diameter are presented in Figure 4. With an increase in temperature from 650 °C to 950 °C, SPS has influenced the relative density and porosity of FMZ (Table 2 and Figure 3).

**Figure 5:**
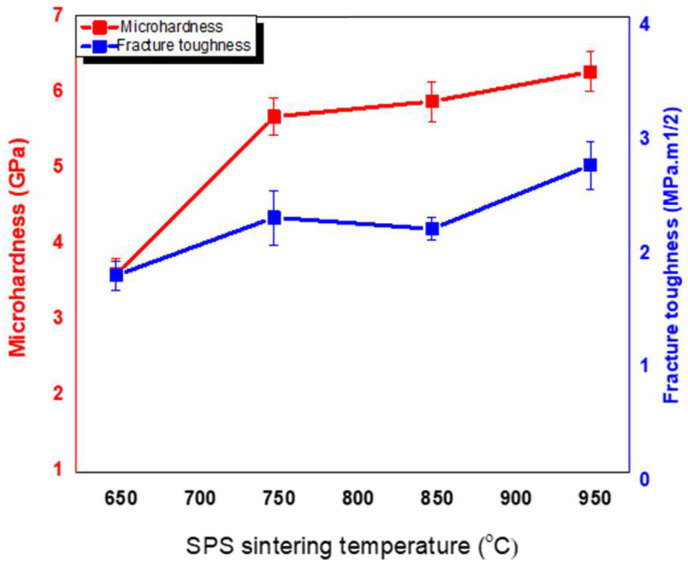
Effect of sintering temperature on micro-hardness and fracture toughness of SPS-FMZ.

**Figure 6:**
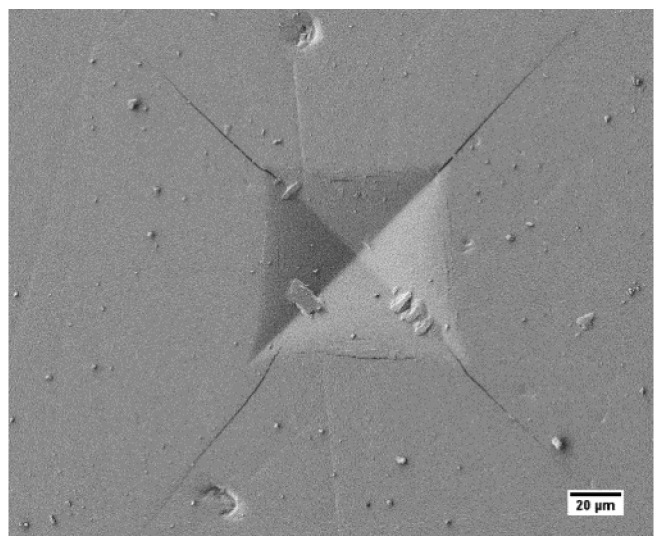
Cracks emanating from Vickers indentation fracture toughness test.

The mechanical properties of SPS-FMZ are presented in Table 3. Figure 5 depicts the effect of SPS sintering temperatures on the hardness of SPS-FMZ and Figure 6 shows the cracks emanating from Vickers indentation. An increase in temperature of SPS-FMZ from 650 °C to 950 °C resulted in higher hardness and fracture toughness values, as shown in Table 3. The hardness values increased with the sintering temperature from 650 °C to 750 °C and almost remained the same until 950 °C, corresponding to the densification values (Table 2 and Table 3). Low hardness values at 650 °C could be corroborated by non-reactive spherical particles with pores, unlike at higher temperatures where agglomerates formed, offering higher resistance to surface indentation. There was not much difference in the hardness values of SPS-FM without YSZ at 950 °C of 5.34 ± 0.06 GPa to the SPS-YSZ at 850 °C of 5.89 ± 0.26 (Table 3).

**Figure 7:**
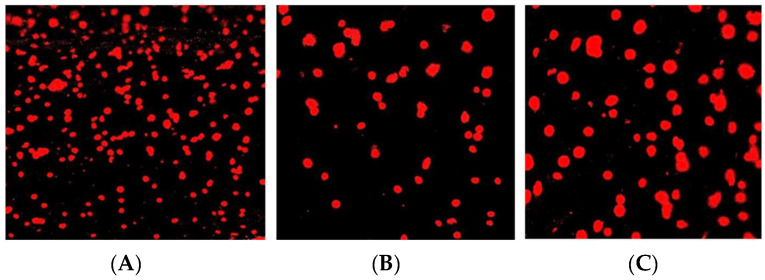
Comet assay images for genotoxicity. (**A**) Negative control, (**B**) IPS e.max glass-ceramic, and (**C**) SPS-FMZ at 950 °C.

The effect of spark-plasma-sintered FM on the nucleus or DNA of human gingival fibroblasts by Comet assay was determined. Comet assay images are presented in Figure 7A–C. With the absence of tail moments in Figure 7, it can be deduced that there was no DNA damage of human gingival fibroblast cell lines from SPS-FMZ and the control, the commercially available IPS e.max glass-ceramic, after 24 h of incubation.

## 4. Discussion

### 4.1. Phase Assemblage

Amorphous glass with minor crystalline phases in the XRD plots of SPS-FMZ could be corroborated by the short holding time (5 min) and low sintering temperatures (650 °C to 950 °C) with a high heating rate of 100 °C/min. Similar amorphous glassy phases were observed in SPS-sintered Bioglass, calcium aluminosilicate oxynitride glass, and sol–gel-based zirconia–silica glass systems [55,56,57,58]. In contrast to SPS, conventionally sintered FMZ at 1080 °C for 48 h at a heating rate of 10 °C/min showed the formation of fluorophlogopite crystals due to the long sintering time that allowed for nucleation and crystallization of FM-based glass [5,18,40]. In addition to the long sintering times and slow heating rates, the amount and source of fluorine as a nucleating agent in FM influence the phase formation and crystallization of fluorophlogopite [5]. As elemental analysis of sintered SPS-FMZ reveals the presence of fluorine (wt.%), high SPS temperatures with heating rates less than 100 °C/min and extended dwell times must be further investigated to initiate crystallization in SPS-FMZ glass [59].

### 4.2. Microstructural Characterization

The spark discharge in SPS between the particles results in high temperature, vaporization, and melting of the powder surfaces, forming a neck between the particles. The formation of necks between the particles could be attributed to the periodic electron current and vacuum with surface heating of the particles [27]. Glass powders typically sinter through a viscous flow mechanism. A slow rate of nucleation and crystallization with two-stage heat treatment characterizes viscous flow in glass ceramics [59]. However, the sintering of glass during the SPS procedure differs and involves densification. The SPS of glass occurs through viscous flow and growth of the neck of the glass particles. Compact glass powders tend to reduce their surface energies through neck growth. An increase in temperature from 650 °C to 950 °C would have enabled the glass’s viscous flow with the glass particles’ necking, as seen in Figure 2A–D. Further, pressure applied during SPS increases the glass viscosity and suppresses the crystal growth [41,60,61]. Restricted crystal growth in SPS promotes high densities [62].

### 4.3. Density and Porosity

The fast heating rates and uniaxial pressure in SPS typically aid in the densification of the glass powders. Densification in SPS occurs through diffusional mass transport that promotes bonding between the particles and the consolidation of powders. Applying pressure and heat transfer from the die with high heating and cooling rates enables densification. Further, using direct current pulses results in an increased diffusion rate. Compressive stress or the pressure applied in SPS results in better contact between powder particles and promotes densification by removing large pores [62]. In SPS, higher pressure can be used to reach high densities in less time and at low temperatures [27,30,52,62]. Under intense pressure, the atoms in glasses rearrange, transitioning from brittle behavior to ductile or plastic deformation. During the SPS of FMZ, the pressure of 50 MPa and the increase in SPS temperatures (from 650 to 950 °C) accompanied by the viscous flow of the glass would have facilitated high densification with reduced porosity percentage and pore size. The addition of YSZ to FM seems to enhance the density of SPS-FM from 1.63 g/cc to 2.97 g/cc (Table 2). Interestingly, some studies reported the crystalline nature of YSZ and the formation of YSZ agglomerates in the glass matrix, inhibiting the densification and crystallization of conventionally sintered mica-based and SPS lithium silicate glasses [20,43].

Porosity is a fundamental parameter determining the suitability of dental ceramics. A porous microstructure of a dental material acts as a stress concentrator and scatters light, lowering its ability to endure masticatory forces with poor esthetics. Porosity is influenced by sintering time, temperature, and sintering atmosphere. The reduction in porosity is advocated by long sintering times and moderately high sintering temperatures [63]. The porosity of previous studies on SPS zirconia-toughened alumina ceramics for dental applications revealed a decrease in porosity and pore size with an increase in temperature [57]. Figure 3 presents pore size distribution plots at sintering temperatures of 650–950 °C, Figure 4 shows the effect of sintering temperature on density–porosity, and Table 2 shows the pore size diameter range of each of the sintered specimens at 650–950 °C, respectively. With the increase in temperature from 650 °C to 950 °C, the mean pore size of the sintered specimens reduced with the shrinking of the pore sizes and reduction in the porosity percentage (Table 2). A decrease in percentage porosity from 42% at 650 °C to 12% at 950 °C could be correlated with improved densities of SPS-FMZ with an increase in temperature. The mean pore diameter of SPS-FMZ specimens sintered from 650 °C to 850 °C reduced from 14 nm to 10 nm with a decrease in pore size range, indicating reduced pore size polydispersity. This observed decrease in the polydispersity pore size range could be due to the formation of uniform pores with increasing temperatures, leading to decreased pore volume and a reduction in percentage porosity. On the other hand, the SPS-FMZ sample sintered at 950 °C exhibits uniform pores (in the range of 6–7 nm) and hence contributes to the closer packing of particles, which is evident from its higher density. It must be noted that although a mercury intrusion porosimeter has the advantages of measuring a wide range of pore sizes and simplicity, it has the limitations of measuring only open pores and excluding closed or bottle-neck-shaped pores.

### 4.4. Mechanical Characterization

The fracture toughness values of SPS-FMZ increased with sintering temperature (Figure 5) and reached a maximum value of 2.7 MPa m^1/2^ at 950 °C. This value was almost twice that of SPS-FM without YSZ, which was 1.22 MPa m^1/2^. Thus, the enhanced fracture toughness values of SPS-FMZ at 950 °C could be attributed to the phase transformation of zirconia shown in Figure 6. Fracture toughness values of SPS-FMZ at 950 °C are comparably higher than conventionally sintered Bioverit glass reinforced with 20 wt.% of pure zirconia particles and commercial pre-crystalline machinable glass-ceramics [18,21]. Conventionally sintered zirconia-reinforced FM glass-ceramics have shown fracture toughness values ranging from 0.8 MPa m^1/2^ to 1.44 MPa m^1/2^ [20]. Fracture toughness is an important clinical property required for dental ceramics more than strength, as it measures the innate ability of the material to resist crack propagation [18].

### 4.5. Genotoxicity

Screening assays of newly developed dental materials are recommended to detect their possible mutagenic and genotoxic potential [64]. Some of the suggested assays are the Ames and Comet assays. The Comet assay typically detects DNA damage of cells through the tailing of the comet through the technique of electrophoresis [65,66,67,68,69]. The Comet assay evaluates the genotoxicity of dental composites, bleaching agents, dental ceramics, polymethyl methacrylate resins, pulp capping, and dentin bonding agents [70,71]. The International Workshop on Genotoxicity Test Procedures recommends visual scoring of cells based on the tail length and classifies them into five classes: class 0, undamaged cells having no tail; class 1, cells having a tail shorter than the diameter of the head (nucleus); class 2, cells having a tail length 1–2 times the diameter of the head; class 3, cells having a tail longer than two times the diameter of the head; and class 4, comets having no heads [71]. The effect of spark-plasma-sintered FM on the nucleus or DNA of human gingival fibroblasts was determined by Comet assay.

In translational research and the development of biomaterials, evaluating dental materials’ biocompatibility is crucial. ISO Standard 10993 Part 3 addresses specific biological tests required for genotoxicity [71,72]. With the absence of tail moments in Figure 7, it can be deduced that there was no DNA damage of human gingival fibroblast cell lines from SPS-FMZ and the control, the commercially available IPS e.max glass-ceramic, indicating its cytocompatible nature due to its non-toxic effect on human gingival fibroblast cells. However, the long-term degradation and chemical durability of SPS-FMZ must be further investigated. Development of dental ceramics requires evaluation of clinically pertinent properties ranging from physical, mechanical, thermal, biocompatibility, and fabrication-related characterizations. The dental restorative ceramics must typically exhibit a fracture toughness of greater than 1.5 MPa m^1/2^ and a chemical solubility of less than 50 mg/cm^2^ with an optimal translucency similar to teeth [2]. In the present study, SPS-FMZ at 950 °C showed an optimal fracture toughness of 2.7 MPa m^1/2^ and a hardness of 6.28 GPa with the absence of genotoxicity in human gingival fibroblast cell lines.

The observed greyish discoloration of SPS-FMZ (inset image in Figure 2F), could be attributed to the carbon contamination from graphite dies and the formation of oxygen vacancies of Si-O-Si and YSZ in the FM glass [73,74,75,76,77]. Similar greyish discoloration has been reported in SPS-zirconia-bearing lithium silicate glass-ceramics for dental restorations [46]. The greyish discoloration could be explored for the dental ceramic cores. Typically, an opaque tougher core with esthetic layering is one of the material options for dental ceramic restorations. This choice is usually indicated for discolored teeth, as opaque cores tend to mask the dark color of the tooth. [78]. Further investigations must be conducted on strategies to reduce the associated discoloration for broader clinical indications. The reported strategies include pre-compacting the glass powders below their glass transition temperatures followed by SPS, the use of carbon-diffusion barriers such as tantalum or molybdenum or platinum foils, and annealing post-SPS sintering [44,46].

## 5. Conclusions and Future Outlook

Based on the results, the following conclusions could be drawn:

- Increase in SPS temperatures from 650 °C to 950 °C at a pressure of 50 MPa with a heating rate of 100 °C/min of 20 wt.% YSZ in fluormica glass retained tetragonal and monoclinic peaks of YSZ with amorphous glass.
- SPS-FMZ at 950 °C resulted in a microstructure of spherical agglomerates with particle coalescence.
- SPS with an increase in temperature improved the density and reduced the porosity of FMZ.
- The fracture toughness and hardness of SPS-FMZ were comparable to the properties required for a dental restorative material.
- There was an absence of genotoxicity of SPS-FMZ in human gingival fibroblast cells, indicating their cytocompatible nature in the oral cavity.
- YSZ played a role in enhancing the fracture toughness and densification of SPS FM glass.

The associated discoloration of SPS-FMZ could be reduced with the use of molybdenum liners and annealing to simulate the optical properties of teeth. Further, relevant properties such as chemical durability, machinability, and processing of slow heating rates with extended sintering times during SPS can be investigated. SPS is an emerging sintering technique that has the potential for the processing of dental restorations with dense microstructures and tailor-made clinically relevant properties. SPS enables functionally graded systems with varying mechanical properties. These graded systems with nanoscale microstructures provide damage-resistant and wear-resistant load-bearing ceramics. The fast sintering in SPS supports rapid crystallization with short holding times at low temperatures [27,28,29,30,31]. The sintering technique thus bids microstructural benefits in terms of wear, strength, toughness, and machinability required for dental ceramic restorations.

## Figures and Tables

**Table 1:** Chemical composition of glass precursors.

Starting Materials	Source	Lot No.	Oxide Constituent	wt.%
Silica gel	Loba, Jehangir Villa, India (400–700 mesh)	5699H	SiO_2_	44.5
Aluminum oxide Active	Loba (Neutral)	00950	Al_2_O_3_	16.7
Magnesium carbonate	Sigma Aldrich, St. Louis, MO, USA (99.5% AR)	227668	MgO	14.5
Potassium carbonate anhydrous	Loba (99.5% AR/ACS)	05336	K_2_O	9.5
Ammonium fluoride	Loba (98% Ar/ACS)	01200	F	6.3
Boric acid crystal (granular)	Loba (99.5% Extra pure)	00218	B_2_O	8.5

**Table 2:** Density values with percentage porosity of SPS-FMZ.

SPS-FMZ	Apparent Density (g/cc)	Percentage Porosity (%)	Mean Pore Size Diameter (nm) ± SD	Pore Diameter Range (nm)
650 °C	2.36 ± 0.11	42	14 ± 11.4	3–51
750 °C	2.79 ± 0.05	35	11.5 ± 4.1	4–18
850 °C	2.84 ± 0.05	18	10.7 ± 4.1	4–18
950 °C	2.97 ± 0.13	12	7.3 ± 1.9	4–10
SPS-FM without YSZ at 950 °C	1.63 ± 0.0	-		

**Table 3:** Mechanical properties of SPS-FMZ. (Dissimilar superscripts (^a^, ^b^, and ^c^) indicate statistically significant difference.)

SPS-FMZ	Micro-Hardness (GPa)	Nano-Hardness (GPa)				Reduced Elastic Modulus (GPa)				Fracture Toughness (MPa m^1/2^)
	Mean ± S.D.	Median	IQR	Min	Max	Median	IQR	Min	Max	Mean ± S.D.
650 °C	3.61 ± 0.21 ^a^	4.93 ^a^	5.42	3.06	12.39	93.79 ^a^	20.76	74.76	173.00	1.73 ± 0.13 ^a^
750 °C	5.69 ± 0.24 ^b^	10.02 ^b^	1.37	7.70	18.94	98.86 ^a^	47.02	38.34	192.62	2.24 ± 0.24 ^a^
850 °C	5.89 ± 0.26 ^c^	11.03 ^b^	7.24	6.47	63.39	121.56 ^b^	6.59	107.80	158.57	2.14 ± 0.10 ^a^
950 °C	6.28 ± 0.26 ^b,c^	11.22 ^b^	2.72	8.40	20.27	112.94 ^b^	14.03	103.45	170.60	2.70 ± 0.21 ^b^
SPS-FM without YSZ at 950 °C	5.34 ± 0.06	7.29	0.96	6.13	9.51	80.48	4.33	74.96	86.79	1.22 ± 0.13

## Data Availability

Not applicable.
